# Integration of real-time patient vital signs into an electronic transport checklist to improve clinical decision-making and checklist responsiveness

**DOI:** 10.1097/MD.0000000000049890

**Published:** 2026-07-31

**Authors:** Mei Li, Qichen He, Xuexin Shen

**Affiliations:** a Department of Emergency Critical Care Medicine, Fuzhou University Affiliated Provincial Hospital, Fuzhou City, Fujian Province, China; b Department of Gastric Surgery, Fujian Medical University Union Hospital, Fuzhou City, Fujian Province, China; c Department of Hepatobiliary and Pancreatic Surgery, Fuzhou University Affiliated Provincial Hospital, Fuzhou City, Fujian Province, China.

**Keywords:** clinical decision-making, critical care, digital health, electronic checklist, intrahospital transport, patient safety, real-time vital signs

## Abstract

This study aimed to evaluate whether integrating real-time vital signs into an electronic intrahospital transport checklist improves abnormal vital sign recognition time, clinical intervention response time, checklist completion efficiency, and transport-related safety outcomes. This prospective, before-and-after (quasi-experimental) observational study was conducted at a tertiary teaching hospital between January 2023 and December 2024. Adult patients requiring intrahospital transport with continuous physiological monitoring were included. Patients transported before June 2023 were managed using a conventional paper-based checklist, while those transported after implementation were managed using an electronic checklist integrated with real-time vital sign monitoring. Primary outcomes included abnormal vital sign recognition time, clinical intervention response time, and checklist completion time. Secondary outcomes included transport-related adverse events, checklist completion rate, and staff satisfaction. A total of 102 patients were included (49 conventional checklist group, 53 electronic checklist group). Baseline characteristics, including illness severity and transport complexity, were comparable between groups. The electronic checklist group demonstrated significantly shorter abnormal vital sign recognition time (16.9 ± 7.3 vs 34.6 ± 11.8 seconds, *P* < .001), faster clinical intervention response time (61.3 ± 21.7 vs 88.7 ± 26.4 seconds, *P* < .001), and reduced checklist completion time (97.2 ± 27.8 vs 126.4 ± 32.5 seconds, *P* < .001). Transport-related adverse events occurred less frequently in the electronic checklist group (9.4% vs 16.3%), although this difference was not statistically significant (*P* = .38). Staff satisfaction scores were significantly higher in the electronic checklist group (4.3 ± 0.6 vs 3.5 ± 0.8, *P* < .001). Integration of real-time vital signs into an electronic intrahospital transport checklist was associated with improved clinical responsiveness, faster intervention, and enhanced workflow efficiency. Given the before-and-after design, mixed timing methods, and limited power for safety endpoints, these findings should be interpreted as process-improvement associations rather than definitive causal evidence. Larger multicenter studies are needed to confirm whether these improvements translate into significant reductions in transport-related adverse events.

## 1. Introduction

Intrahospital transport of critically ill patients is an essential component of modern hospital care, allowing access to diagnostic imaging, surgical procedures, and specialized therapeutic interventions. However, intrahospital transport is widely recognized as a high-risk process associated with physiological instability and adverse clinical events. Previous studies have reported adverse event rates ranging from 5 to 70%, including hypoxemia, hypotension, arrhythmias, accidental extubation, and equipment-related complications.^[1]^ These adverse events can significantly increase morbidity, prolong hospitalization, and increase healthcare costs. Furthermore, even minor physiological deterioration during transport may lead to serious clinical consequences if not promptly recognized and treated.^[2]^ Therefore, ensuring patient safety during intrahospital transport remains a critical priority in clinical practice.

Delayed recognition of physiological deterioration is one of the major contributors to transport-related adverse events. Critically ill patients often require continuous monitoring and rapid clinical intervention to maintain physiological stability. However, during intrahospital transport, clinicians must simultaneously manage monitoring equipment, ensure device functionality, and complete necessary documentation. This complex workflow increases cognitive workload and may delay the timely recognition of abnormal vital signs.^[3]^ Continuous physiological monitoring plays a crucial role in detecting early signs of clinical deterioration, allowing clinicians to intervene before complications worsen.^[4]^ Early identification of abnormal vital signs has been associated with improved clinical outcomes and reduced adverse event rates.

Transport checklists have been widely recommended as an effective strategy to standardize care and improve patient safety during intrahospital transport. Checklists serve as cognitive aids that help clinicians ensure that essential safety steps are completed before and during transport. Previous studies have demonstrated that transport checklists improve preparation quality, reduce omissions, and decrease transport-related complications.^[5]^ The use of checklists has been associated with improved clinical outcomes across various healthcare settings, including surgery, intensive care, and emergency medicine.^[6]^ Despite their benefits, traditional transport checklists are typically paper-based and rely on manual observation and documentation. This manual process can be time-consuming and may limit the checklist’s effectiveness in rapidly changing clinical environments.

Advances in healthcare information technology have facilitated the development of electronic checklist systems, which offer several advantages over traditional paper-based methods. Electronic checklists can improve accessibility, enhance documentation accuracy, and integrate with electronic health records (EHRs).^[7]^ More importantly, electronic checklist systems can integrate real-time physiological monitoring data, such as heart rate, blood pressure, oxygen saturation, and respiratory rate. This integration allows clinicians to continuously monitor patient status and receive immediate alerts when abnormal vital signs occur, thereby improving situational awareness and reducing response delays.

Electronic early warning systems and automated monitoring technologies have demonstrated significant benefits in improving the early detection of patient deterioration. Studies have shown that electronic monitoring systems can enhance clinicians’ ability to detect physiological abnormalities and improve response times.^[8]^ Automated alert systems based on real-time physiological data have been associated with improved patient safety and reduced adverse events in hospitalized patients.^[9]^ These systems support clinical decision-making by providing timely and accurate information, which is essential for managing critically ill patients.

Intrahospital transport presents unique challenges compared with other clinical environments. The physical movement of patients, changes in monitoring conditions, and potential equipment disconnections may increase the risk of physiological instability. In addition, clinicians must make rapid clinical decisions based on continuously changing physiological information. Integrating real-time vital signs into an electronic transport checklist may improve checklist responsiveness, enhance clinical decision-making, and reduce transport-related adverse events. Electronic systems may also improve workflow efficiency and reduce manual workload by automating data capture and providing visual alerts.^[10]^

Despite the theoretical advantages of integrating real-time vital signs into electronic transport checklists, clinical evidence evaluating their effectiveness remains limited. Therefore, the aim of this study was to evaluate whether integrating real-time vital signs into an electronic intrahospital transport checklist improves abnormal vital sign recognition time, clinical intervention response time, checklist completion efficiency, and transport-related patient safety outcomes. We hypothesized that electronic checklist integration would significantly improve clinical responsiveness and reduce transport-related adverse events compared with conventional paper-based checklists.

## 2. Methods

### 2.1. Study design and setting

This study was approved by the Ethics Committee of Fuzhou University Affiliated Provincial Hospital. This prospective, before-and-after observational study was conducted at a tertiary teaching hospital between January 2023 and December 2024. The hospital has more than 1500 beds and provides comprehensive critical care, emergency, and surgical services. The electronic intrahospital transport checklist system was implemented in June 2023 and progressively adopted across clinical departments following system deployment and staff training. Patients transported before implementation were managed using the conventional paper-based checklist, whereas patients transported after implementation were managed using the electronic checklist system integrated with real-time vital sign monitoring.The study protocol was reviewed and approved by the IRB of the hospital. The requirement for written informed consent was waived due to the observational nature of the study and the use of routinely collected clinical data. All data were anonymized prior to analysis.

Because group allocation followed the institutional implementation timeline rather than patient-level randomization, this study should be interpreted as a prospective before-and-after quasi-experimental evaluation and as hypothesis-generating. Randomization was not feasible because the electronic checklist was deployed at the department/workflow level and subsequently became part of routine intrahospital transport practice. To reduce confounding, we collected and compared variables reflecting patient severity and transport complexity, including Sequential Organ Failure Assessment (SOFA) score, mechanical ventilation, vasopressor use, continuous sedation infusion, transport origin, destination, and transport duration. Nevertheless, baseline comparability cannot eliminate unmeasured confounding, temporal trends, Hawthorne effects, or changes in team familiarity after implementation; these issues are explicitly addressed in the Discussion.

### 2.2. Participants

All adult patients requiring intrahospital transport during the study period were screened for eligibility.

#### 2.2.1. Inclusion criteria

Patients were eligible for inclusion if they met the following criteria: Age ≥ 18 years; required intrahospital transport from the intensive care unit, emergency department, or general ward to diagnostic or treatment areas, including computed tomography (CT), magnetic resonance imaging (MRI), or the operating room (OR); required continuous physiological monitoring during transport; transport duration ≥ 5 minutes.

#### 2.2.2. Exclusion criteria

Patients were excluded if they met any of the following criteria: incomplete physiological monitoring data during transport, transport duration < 5 minutes, cardiac arrest prior to transport, incomplete checklist documentation. A total of 109 patients were screened. Seven patients were excluded, and 102 patients were included in the final analysis.

### 2.3. Group allocation

Patients were assigned to study groups based on the checklist system used during intrahospital transport, corresponding to the implementation timeline of the electronic checklist system. The conventional checklist group included patients transported between January 2023 and May 2023, during which the conventional paper-based transport checklist was used. This checklist required manual documentation of patient identification, vital signs, equipment preparation, and safety verification.The electronic checklist group included patients transported between June 2023 and December 2024, following implementation of the electronic intrahospital transport checklist system. The electronic checklist was integrated with the physiological monitoring system and automatically captured real-time patient vital signs, including heart rate, blood pressure, oxygen saturation, and respiratory rate. Physiological parameters were continuously displayed on the electronic checklist interface during transport, and abnormal values triggered visual alerts to assist clinicians in identifying physiological deterioration.

### 2.4. Electronic checklist system and real-time data integration

The electronic checklist was embedded in the hospital information platform and linked to the portable physiological monitoring system through the hospital network gateway. The checklist interface displayed patient identification, transport destination, pre-transport equipment and oxygen checks, medication and vascular-access confirmation, a dynamic vital-sign panel, abnormal-event prompts, and an intervention confirmation log. Heart rate, oxygen saturation, and respiratory rate were refreshed on the checklist interface every 5 seconds; noninvasive blood pressure was updated immediately after each configured monitor measurement. All data elements were time-stamped using hospital server time. Visual alerts were generated when predefined abnormal thresholds were sustained for at least 10 seconds, corresponding to at least 2 consecutive monitor updates. The system flagged missing signals, poor signal quality, and possible sensor disconnection; these events required bedside confirmation by the transport team and were reviewed before analysis. The electronic checklist was intended to support, not replace, clinical judgment.

### 2.5. Transport procedure

All intrahospital transports were conducted according to institutional transport protocols. Each transport team consisted of at least one registered nurse and one physician or trained transport personnel. Patients were continuously monitored using portable physiological monitors throughout transport. Clinicians completed either the paper-based checklist or the electronic checklist before and during transport. Clinical interventions were performed as needed based on patient condition and institutional protocols.

### 2.6. Outcome measures

The primary outcomes included abnormal vital sign recognition time, clinical intervention response time, and checklist completion time. Abnormal vital sign recognition time was defined as the time interval, measured in seconds, between the onset of the first qualifying abnormal vital sign event and clinician recognition of the abnormality. Clinical intervention response time was defined as the time interval, measured in seconds, between abnormal vital sign onset and initiation of the first corresponding clinical intervention. Checklist completion time was defined as the total time required to complete the transport checklist. Abnormal vital signs were defined as values exceeding predefined thresholds and sustained for at least 10 seconds, corresponding to at least 2 consecutive monitor updates. Thresholds included oxygen saturation < 90%, systolic blood pressure < 90 mm Hg, heart rate < 50 beats per minute or >130 beats per minute, and respiratory rate < 8 breaths per minute or >30 breaths per minute. To reduce false-positive events caused by patient movement, sensor displacement, or signal loss, events associated with obvious sensor disconnection, missing signals, or poor signal quality were excluded after review of monitor trend data and transport records. When multiple abnormalities occurred during a transport episode, only the first qualifying event was used for analysis. Clinician recognition was defined as the first documented Acknowledgments of abnormal vital signs. In the electronic checklist group, recognition and intervention times were automatically recorded by the electronic system. In the conventional checklist group, recognition and intervention times were recorded by a trained independent observer using a synchronized stopwatch and verified against checklist documentation.Secondary outcomes included transport-related adverse events, checklist completion rate, and staff satisfaction. Transport-related adverse events were defined as hypoxemia (oxygen saturation < 90%), hypotension (systolic blood pressure < 90 mm Hg), arrhythmia requiring intervention, or cardiac arrest during transport.Checklist completion rate was defined as the proportion of transports with complete checklist documentation. Staff satisfaction was assessed using a standardized questionnaire based on a five-point Likert scale (1 = very dissatisfied, 5 = very satisfied), completed by transport staff immediately after transport. To minimize information and measurement bias, abnormality onset was determined from monitor trend records rather than from staff recall in both groups. Observer timing devices, monitor clocks, and the electronic checklist server were synchronized daily with hospital standard time. In the conventional group, observers were trained research personnel who did not participate in patient management and used standardized event definitions and a synchronized stopwatch. Recognition and intervention times were verified against transport documentation and monitor trend data whenever possible. In the electronic group, system time stamps were automatically generated when alerts and intervention confirmations were logged. Because automatic time capture may be more precise than manual observation, residual measurement bias could not be fully excluded and is acknowledged as a methodological limitation.

### 2.7. Data collection

Data were collected prospectively by trained research nurses using standardized data collection forms. Collected variables included patient demographic characteristics, transport origin, transport duration, physiological monitoring data, abnormal vital sign recognition time, intervention response time, checklist completion status, adverse events, and staff satisfaction scores. Additional baseline variables were collected to characterize illness severity and treatment intensity, including vasopressor use during transport, continuous sedation infusion during transport, transport destination (CT, MRI, or OR), and illness severity assessed using the SOFA score immediately prior to transport. In the electronic checklist group, physiological data, abnormal event alerts, and clinical response times were automatically recorded in the electronic system. In the conventional checklist group, data were recorded by trained observers and verified using checklist documentation.All observers received standardized training prior to study initiation. Transport monitor clocks, electronic system clocks, and observer timing devices were synchronized daily using hospital standard time to ensure accuracy and consistency. Before study initiation, data collectors received standardized training on abnormal vital-sign thresholds, stopwatch operation, documentation rules, and handling of signal artifacts. For ambiguous events, 2 investigators reviewed de-identified trend data and transport records to determine whether the event met the predefined criteria. This adjudication was performed before statistical comparison of group outcomes.

### 2.8. Statistical analysis

Statistical analysis was performed using SPSS version 27.0 (IBM Corp., Armonk). Continuous variables were expressed as mean ± standard deviation and compared using independent-samples *t*-tests. Categorical variables were expressed as frequencies and percentages and compared using chi-square tests or Fisher exact tests, as appropriate. All statistical tests were two-tailed, and a *P*-value < .05 was considered statistically significant.

Because this evaluation was embedded in routine implementation, a formal a priori sample size calculation was not completed before system deployment. To address this limitation, we conducted a supplementary sample size verification based on the 3 prespecified primary process outcomes. For 2 independent groups, sample size was estimated using a two-sided independent-samples *t*-test (α = 0.05, power = 80%, allocation ratio approximately 1:1), with Cohen *d* calculated from the observed between-group difference divided by the pooled standard deviation. The most conservative primary outcome was checklist completion time (126.4 ± 32.5 vs 97.2 ± 27.8 seconds; Cohen *d* = 0.97), which required approximately 18 patients per group. After allowing for approximately 10% incomplete or unusable records, the minimum target sample size was 20 patients per group. The final analyzed sample included 49 patients in the conventional group and 53 patients in the electronic checklist group, exceeding the calculated requirement; therefore, the study sample size was adequate for detecting the observed differences in the primary process outcomes. For comparison, the required sample sizes for abnormal vital sign recognition time (Cohen *d* = 1.80) and clinical intervention response time (Cohen *d* = 1.13) were approximately 6 and 14 patients per group, respectively. In contrast, detecting the observed difference in transport-related adverse events (16.3% vs 9.4%) would require approximately 360 patients per group; therefore, the present study was not powered for definitive safety-event conclusions, and adverse-event findings were considered exploratory.

### 2.9. Ethics approval and data privacy

This study was reviewed and approved by the Institutional Review Board (IRB) of Fuzhou University Affiliated Provincial Hospital. Due to the observational nature of the study and the use of routinely collected clinical data, the requirement for written informed consent was waived by the IRB. All study procedures were conducted in accordance with the ethical standards of the institutional and national research committees and with the 1964 Helsinki Declaration and its later amendments or comparable ethical standards. To protect patient privacy and confidentiality, all collected data were anonymized prior to analysis. Patient identifiers, including names, identification numbers, and contact information, were removed before data processing. Electronic data were stored on secure, password-protected hospital servers accessible only to authorized research personnel. No identifiable patient information was used in analysis or publication.

## 3. Results

### 3.1. Participant characteristics

A total of 109 patients were screened between January 2023 and December 2024. Seven patients were excluded due to incomplete monitoring records or transport duration <5 minutes. A total of 102 patients were included in the final analysis, including 49 patients in the conventional checklist group and 53 patients in the electronic checklist group.

Baseline demographic and clinical characteristics are presented in Table 1. There were no statistically significant differences between groups in age, sex, ICU origin, transport duration, mechanical ventilation status, vasopressor use during transport, continuous sedation infusion, or pre-transport SOFA score (all *P* > .05). Transport destination distribution (CT/MRI/OR) was also comparable between groups (χ^2^ = 0.20, *P* = .91). These findings indicate that the 2 groups were comparable in baseline characteristics, illness severity, treatment intensity, and transport complexity.

**Table 1:** Baseline demographic and clinical characteristics.

Variable	Conventional (n = 49)	Electronic (n = 53)	*t*/χ^2^	*P*-value
Age (yr), mean ± SD	59.8 ± 14.7	61.1 ± 13.2	0.48	.63
Male, n (%)	28 (57.1)	32 (60.4)	0.12	.74
ICU origin, n (%)	31 (63.3)	35 (66.0)	0.08	.78
Transport duration (min), mean ± SD	17.9 ± 6.8	18.6 ± 7.1	0.55	.58
Mechanical ventilation, n (%)	21 (42.9)	25 (47.2)	0.19	.66
Vasopressor use during transport, n (%)	14 (28.6)	16 (30.2)	0.03	.86
Continuous sedation infusion, n (%)	18 (36.7)	17 (32.1)	0.24	.62
Transport destination (CT/MRI/operating room), n (%)				
CT	28 (57.1)	31 (58.5)	0.20	.91
MRI	9 (18.4)	8 (15.1)		
Operating room	12 (24.5)	14 (26.4)		
SOFA score before transport, mean ± SD	6.2 ± 2.7	6.5 ± 2.5	0.58	.56

CT = computed tomography, MRI = magnetic resonance imaging, SD = standard deviation, SOFA = Sequential Organ Failure Assessment.

### 3.2. Abnormal vital sign recognition time

Abnormal vital sign recognition time was significantly shorter in the electronic checklist group compared with the conventional checklist group (Table 2). The mean recognition time was 34.6 ± 11.8 seconds in the conventional group and 16.9 ± 7.3 seconds in the electronic group (*t* = 9.21, *P* < .001). The supplementary power estimation indicated that substantially larger cohorts would be required to confirm whether this numerical reduction in adverse events represents a true safety benefit. Therefore, adverse-event results were considered exploratory rather than confirmatory.

**Table 2:** Abnormal vital sign recognition time.

Outcome	Conventional	Electronic	Mean difference	*t*	*P*
Recognition time (s)	34.6 ± 11.8	16.9 ± 7.3	17.7	9.21	<.001

### 3.3. Clinical intervention response time

Clinical intervention response time was also significantly shorter in the electronic checklist group (Table 3). The mean response time was 88.7 ± 26.4 seconds in the conventional group and 61.3 ± 21.7 seconds in the electronic group (*t* = 5.74, *P* < .001).

**Table 3:** Clinical intervention response time.

Outcome	Conventional	Electronic	Mean difference	*t*	*P*
Response time (s)	88.7 ± 26.4	61.3 ± 21.7	27.4	5.74	<.001

### 3.4. Checklist completion time

Checklist completion time was shorter in the electronic checklist group (Table 4). The mean completion time was 126.4 ± 32.5 seconds in the conventional group and 97.2 ± 27.8 seconds in the electronic group (*t* = 4.82, *P* < .001).

**Table 4:** Checklist completion time.

Outcome	Conventional	Electronic	Mean difference	*t*	*P*
Completion time (s)	126.4 ± 32.5	97.2 ± 27.8	29.2	4.82	<.001

### 3.5. Transport-related adverse events

Transport-related adverse events occurred in 8 patients (16.3%) in the conventional checklist group and 5 patients (9.4%) in the electronic checklist group. Although the incidence of adverse events was lower in the electronic checklist group, the difference was not statistically significant (Fisher exact test, *P* = .38) (Table 5). The most common adverse events were hypoxemia and hypotension. No cases of cardiac arrest occurred in either group during transport.

**Table 5:** Transport-related adverse events.

Adverse event	Conventional (n = 49)	Electronic (n = 53)
Any adverse event, n (%)	8 (16.3%)	5 (9.4%)
Hypoxemia, n	3	2
Hypotension, n	3	2
Arrhythmia, n	2	1
Cardiac arrest, n	0	0

Note: Fisher exact test, *P* = .38.

### 3.6. Checklist completion rate and staff satisfaction

Checklist completion rate was higher in the electronic checklist group compared with the conventional checklist group, although the difference did not reach statistical significance (98.1% vs 91.8%; Fisher exact test, *P* = .19). Staff satisfaction scores were significantly higher in the electronic checklist group than in the conventional checklist group (4.3 ± 0.6 vs 3.5 ± 0.8; *t* = 5.71, *P* < .001), indicating improved usability and clinician acceptance of the electronic checklist system (Table 6).

**Table 6:** Checklist completion rate and staff satisfaction.

Outcome	Conventional (n = 49)	Electronic (n = 53)	Test	*P*-value
Checklist completion rate, n (%)	45 (91.8%)	52 (98.1%)	Fisher exact test	.19
Staff satisfaction score (1–5), mean ± SD	3.5 ± 0.8	4.3 ± 0.6	Independent-samples *t*-test	<.001

SD = standard deviation.

## 4. Discussion

This prospective, before-and-after observational study evaluated whether integrating real-time vital signs into an electronic intrahospital transport checklist could improve clinical responsiveness and workflow efficiency compared with a conventional paper-based checklist. The principal findings were that the electronic checklist group demonstrated substantially shorter abnormal vital sign recognition time, faster clinical intervention response time, and reduced checklist completion time, while showing a numerically lower (but not statistically significant) rate of transport-related adverse events. Together, these results support the clinical value of embedding continuously updated physiological data directly within the transport safety workflow.

A key contribution of this work is that it operationalizes the long-standing recommendation that transport safety tools should be process-oriented, phase-specific, and aligned with real-world transport hazards.^[11]^ Prior checklist development frameworks emphasize that transport-related risk is not limited to equipment readiness; it also includes dynamic clinical instability and the need for rapid response during movement and transitions.^[11]^ Our findings extend this concept by demonstrating that when the checklist becomes a live interface for real-time patient status – rather than a static documentation form – clinicians appear to detect abnormal physiology earlier and initiate interventions sooner. This is consistent with evidence that structured transport checklists can improve compliance with safety guidelines and reduce omissions, thereby improving transport processes and potentially safety outcomes.^[12]^ The present study suggests that digital transformation of the checklist may further amplify these benefits by reducing the time penalty associated with manual observation and documentation.

The observed reduction in recognition time likely reflects improved situational awareness and reduced information friction. In conventional transport workflows, clinicians must split attention across multiple sources: monitor screens, equipment checks, patient assessment, and paperwork. This creates opportunities for delays in noticing subtle deterioration, particularly under time pressure. Electronic checklist interfaces that display continuously updated vital signs in the same visual field as required safety checks may streamline attention allocation and shorten the interval between physiological change and clinical acknowledgment. Similar advantages have been reported in other high-risk clinical communication tasks, where digital or structured checklists improved completeness and standardization compared with unstructured processes.^[13]^ Although the Verholen study focused on ICU handover rather than intrahospital transport, the underlying mechanism – standardized digital workflows that support task execution and documentation – may be comparable.^[13]^

Our results also align with the broader literature on early warning and real-time deterioration detection. Traditional early warning scores were developed to identify at-risk patients but show variability in methodology quality and implementation evidence.^[14]^ More recent approaches leverage electronic records and continuous data to produce hospital-wide risk estimates. For example, the Hospital-wide Alert and Validation for Early Notification system combined vital signs, laboratory values, and patient factors to identify deterioration risk and demonstrated performance advantages over comparator tools in its development and validation cohort.^[15]^ While our study did not apply predictive modeling, it draws from the same principle: real-time physiological data should be actionable at the point of care, embedded into workflows where rapid decisions must be made. Intrahospital transport is an especially time-sensitive context, and the benefits we observed in recognition and response time suggest that workflow-integrated monitoring may be a pragmatic step toward earlier mitigation of deterioration during transport.

Importantly, integrating real-time monitoring into checklists introduces new challenges that must be addressed to preserve safety gains. Alarm fatigue and signal artifacts are well-recognized problems in continuous monitoring environments, where nonactionable alarms can desensitize clinicians and paradoxically delay responses to true deterioration. Machine learning approaches have been shown to help distinguish true alerts from artifacts in multisignal monitoring streams, offering one pathway to improving alert quality and reducing noise.^[16]^ In parallel, systematic reviews indicate that alarm burden affects nursing work, perception of alarms, and potentially response behaviors, emphasizing the need for careful alarm governance and human-centered design.^[17]^ In the transport setting – where movement, sensor displacement, and intermittent signal loss are common – artifact filtering and contextual alert logic may be particularly important. Therefore, implementation of electronic transport checklists should include technical safeguards (e.g., signal quality indicators), clinical threshold calibration, and training to ensure that alerts remain meaningful and trusted.

The electronic checklist group also demonstrated shorter checklist completion time, which may reflect automated data capture and reduced documentation burden. Documentation time is not merely an efficiency metric; it can influence clinical attention and cognitive workload during high-risk tasks. Evidence suggests that EHR-related workload and interface complexity can contribute to clinician burnout and cognitive overload, highlighting that digital tools must be designed to reduce (not add) cognitive burden.^[18,19]^ In our study, the combination of automatic vital sign capture and on-screen prompts may have reduced manual transcription demands and helped clinicians complete required checks faster. This supports a design principle emphasized in digital health evaluation: technology should demonstrate practical utility, usability, and workflow fit, not only technical capability.^[20]^ From a patient-safety perspective, reducing time spent on paperwork during transport may allow more attention to direct patient observation and proactive management.

The reduction in adverse events did not reach statistical significance, likely due to a limited sample size and the relatively low absolute event count. However, the direction of effect is clinically plausible, and intermediate process improvements (faster recognition and response) are meaningful because they represent mechanisms through which adverse events may be prevented or mitigated. Larger studies are needed to determine whether the improvements in responsiveness translate into measurable reductions in hypoxemia, hypotension, arrhythmias, or escalation of care. Additionally, our outcomes were measured as time intervals from abnormality onset; future work may enrich this by including severity-adjusted outcomes, transport complexity, destination type (CT/MRI/OR), and patient acuity indices.

Several considerations should guide interpretation. First, group allocation was based on checklist system usage and implementation timing rather than randomization. Although measured baseline characteristics, illness severity, treatment intensity, and transport complexity were comparable, a before-and-after design cannot rule out residual confounding from unmeasured patient factors, temporal changes in staffing, safety culture, workflow familiarity, or concurrent quality-improvement activities. Therefore, the observed differences should be interpreted as associations rather than proof of causality. Second, the electronic group relied on automatic timestamps, whereas the conventional group relied on trained observer timing and stopwatch recording. We attempted to mitigate this difference by using standardized definitions, synchronized clocks, independent observers, monitor trend verification, and predefined adjudication rules; however, automatic recording may still provide greater temporal precision than manual observation. This potential information or measurement bias may have influenced the magnitude of the observed time differences. Third, the sample size was sufficient for the large observed process outcomes but not for transport-related adverse events. The nonsignificant adverse-event result should therefore not be interpreted as evidence of no safety benefit. Fourth, the study was conducted at a single tertiary teaching hospital with specific transport protocols, monitoring equipment, network infrastructure, and staff training resources, which may limit generalizability to hospitals with different resource levels. Fifth, staff satisfaction may have been influenced by novelty effects and perceived modernization; longer-term usability evaluation is required to determine whether acceptance is sustained.

Successful promotion of this model requires several conditions: portable monitors capable of digital data output, reliable wireless or wired network coverage along transport routes, an interface between monitors and the electronic checklist/EHR, secure data storage, technical maintenance, alarm-governance rules, and standardized staff training. Training should include checklist operation, interpretation of visual alerts, confirmation of signal quality, response algorithms for hypoxemia, hypotension, arrhythmia, and respiratory deterioration, and downtime procedures for network interruption. The main cost items include hardware interfaces or gateways, software development and maintenance, cybersecurity/data-governance support, user training, and ongoing quality monitoring. In resource-limited environments, full real-time integration may be challenging; a staged implementation may be more feasible, such as using a low-cost tablet-based checklist, manual entry of key vital signs at fixed intervals, selective real-time connection for the highest-risk transports, offline data caching, and simplified threshold-based alerts. Local feasibility should be assessed according to monitor availability, IT support capacity, patient volume, transport risk profile, and staff workload. Because this study did not include a cost-effectiveness analysis, future work should evaluate whether efficiency gains and potential adverse-event reductions justify implementation costs in different hospital settings.

Future research should prioritize multicenter, adequately powered trials and mixed-method implementation evaluations. Digital health reporting frameworks emphasize transparency in describing intervention components, implementation context, and workflow integration. Studies should also explore adaptive alert thresholds tailored to patient baseline status, artifact-aware alert logic, and predictive risk indicators derived from streaming data. Evidence from real-time risk prediction work on hospital wards suggests the feasibility of electronic, continuously updated risk estimates based on EHR and monitoring signals.^[21,22]^ Translating similar approaches into transport settings could enable proactive preparation (e.g., escalation of escort level or equipment readiness) before instability occurs. At the same time, alarm fatigue must be actively monitored and mitigated through technical and organizational strategies, as recent scoping work highlights the complexity of alarm fatigue definitions, measurement, and multi-level interventions.^[23–25]^ Overall, our findings support the concept that transport safety tools should evolve from static checklists to responsive, data-integrated clinical support systems.

## 5. Conclusion

Integrating real-time vital signs into an electronic intrahospital transport checklist was associated with faster recognition of abnormal physiology, shorter intervention response time, and improved checklist efficiency compared with a conventional paper checklist. However, because this was a nonrandomized before-and-after study with different timing methods between groups and limited power for adverse events, the results should be interpreted as preliminary process-improvement evidence rather than definitive causal proof of improved safety outcomes. Although adverse events were numerically lower in the electronic group, larger multicenter studies using standardized outcome measurement are warranted to confirm safety benefits, evaluate cost-effectiveness, and optimize alert design to minimize alarm fatigue.

## Author contributions

**Conceptualization:** Mei Li, Qichen He, Xuexin Shen.

**Data curation:** Mei Li, Qichen He, Xuexin Shen.

**Formal analysis:** Mei Li, Qichen He, Xuexin Shen.

**Funding acquisition:** Mei Li.

**Investigation:** Mei Li.

**Writing – original draft:** Mei Li.

**Writing – review & editing:** Mei Li.
